# Metabolic Changes after Urinary Diversion

**DOI:** 10.1155/2011/764325

**Published:** 2011-05-12

**Authors:** Frank Van der Aa, Steven Joniau, Marcel Van Den Branden, Hein Van Poppel

**Affiliations:** ^1^Department of Urology, University Hospitals Leuven, B-3000 Leuven, Belgium; ^2^AZ Sint-Blasius, 9200 Dendermonde, Belgium

## Abstract

Urinary diversion is performed on a regular basis in urological practice. Surgeons tend to underestimate the metabolic effects of any type of diversion. From the patient's perspective, diarrhea is the most bothersome complaint after urinary diversion. This might be accompanied by malabsorption syndromes, such as vitamin B12 deficiency. Electrolyte abnormalities can occur frequently such as hyperchloremic metabolic acidosis, or less frequently such as hypokalemia, hypocalcaemia, and hypomagnesaemia. Bone health is at risk in patients with urinary diversion. Some patients might benefit from vitamin D and calcium supplementation. Many patients are also subject to urinary calculus formation, both at the level of the upper urinary tract as in intestinal reservoirs. Urinary diversion can affect hepatic metabolism, certainly in the presence of urea-splitting bacteria. The kidney function has to be monitored prior to and lifelong after urinary diversion. Screening for reversible causes of renal deterioration is an integral part of the followup.

## 1. Introduction

In the majority of cases, urinary diversion is performed after cystectomy to treat high-risk nonmuscle invasive bladder cancer after failure of intravesical therapy or to treat muscle invasive bladder cancer. Urinary diversions can be divided in noncontinent diversions, continent diversions, and orthotopic neobladders. Currently, the majority of urinary diversions are constructed from terminal ileum or ileocolonic segments of the intestine. Urologists who perform urinary diversions should not only be familiar with surgical techniques to create these diversions but should also be aware of metabolic changes that arise when intestinal segments are used to divert or to store urine. Many patients have a long life expectancy, even after oncological surgery with urinary diversion. The advance of medical care makes urinary diversion possible in older, less fit patients with impaired renal function. The duration of contact between urine and bowel, the segment and length of bowel used are factors that determine the nature and grade of metabolic effects. Diversion will result in immediate changes in metabolism. Complications can occur soon after diversion. Many complications, however, will only become clear many months or years after the surgical procedure. Therefore, long-term followup and prevention of complications is mandatory. Although diversions have been performed since many decades, many aspects regarding followup and prevention of metabolic changes remain under debate. Good clinical studies are lacking and most recommendations are based on expert opinion and low quality data.

In this paper, we will describe the relevant short, and long-term metabolic changes in urinary diversion using ileal and ileocolonic segments. We will emphasize on clinical followup, treatment of these metabolic changes, and prevention of complications.

## 2. Article

The most popular diversions to date are made from ileal or ileocolonic segments. Noncontinent ileocutaneostomy or Bricker diversion is the most frequently used type of diversion. This procedure was popularized by Bricker [1]. In this procedure, a conduit is made from approximately 15 to 25 centimeters of preterminal ileum. Reasons for this popularity over other types of diversion are the relative ease and simplicity of the procedure, the predictable functional results (no risk for incontinence, retention, and catheterization problems), and the fact that this type of diversion results in less metabolic changes (shorter bowel segment, no urinary storage). Nevertheless, about 10% of patients with ileal conduits will have metabolic disturbances requiring therapy [2]. Several pouches constructed from detubularized ileal segments can be used to create continent diversions or orthotopic neobladders. The W-pouch or Hautmann pouch, the Stüder pouch, the N-pouch, and the Kock pouch are some variants on this theme [3–7]. In contrast to the ileal conduit, 40–80 centimeters of preterminal ileum are used for these types of diversion. The ileal segment is detubularized in order to create a larger, low pressure reservoir. In this way, reservoirs can be made with capacities that are similar to the native bladder. As a consequence, urine will have a long contact time with the intestinal segment, allowing extensive metabolic exchange. Ileocolonic pouches are constructed in a similar way. Instead, terminal ileum together with caecum are detubularized to create a reservoir. One of the most popular examples of these techniques is the Mainz pouch [8, 9]. Metabolic consequences of these pouches are in general comparable to ileal pouches, although some differences exist. Another example of reservoir that uses ileocolonic bowel segments is the Indiana pouch. In fact, this is a detubularized right colonic reservoir that uses a plicated ileal outlet to create a continent cutaneous diversion [10, 11].

## 3. Bowel Dysfunction/Malabsorption

One of the main reasons for diminished quality of life after urinary diversion is diarrhea [6]. Several factors explain the possible occurrence of diarrhea after urinary diversion. Resection of a large part of preterminal ileum results in diminished bile salt and fat absorption. Normally, bile salts are produced in the liver and partially stored in the gall bladder. After food ingestion they are secreted in the duodenum. They emulsify fats and are reabsorbed in the preterminal ileum, entering the enterohepatic cycle. In normal circumstances, 95% of bile salts are recycled. When larger amounts of bile salts reach the colon, they act as mucosal irritants, directly causing diarrhea. Fat malabsorption only occurs when larger portions of small intestine are resected. This results in steatorrhea. Resection of the ileocaecal valve increases the risk to develop bothersome diarrhea. It can result in bacterial overgrowth of the ileum. This reduces the absorptive capacity of this bowel segment, resulting again in bile salt and fat malabsorption, causing diarrhea. Resection of larger parts of colon can also result in diarrhea. When the alkaline ileal content cannot be absorbed by the shortened colonic segment, this results in dehydration and acidosis. Bowel dysfunction is more prevalent in neurogenic patients. In general, nutritional deficiencies are rare since large portions of jejunum are not used for urinary diversions. The treatment of persisting bothersome diarrhea after urinary diversion consists of cholestyramine, a resin that binds bile salts, increased dietary fiber intake (in general in fruits, vegetables, whole grain products). The dose of cholestyramine has to be increased gradually from 4 g b.i.d. to 8 g b.i.d. It has to be taken separately from other medication. Long-term, high-dose use of cholestyramine can also induce deficiency of fat soluble vitamins. If these measures prove to be insufficient, gastrointestinal motility inhibitors such as loperamide 4 Mg q.d. to 16 Mg q.d. can be added. It is best not to advise fluid restriction, since patients with urinary diversion are subject to dehydratation [12].

## 4. Acid Base Abnormalities

A hyperchloremic metabolic acidosis is encountered in all patients that undergo urinary diversion using ileal and/or colonic segments. In the bowel, sodium is secreted in exchange of hydrogen and bicarbonate is secreted in exchange of chloride. In parts of bowel that are exposed to urine, ammonia, ammonium, hydrogen, and chloride are reabsorbed as well. As a consequence, the presence of an ileal and/or colonic urinary diversion always implies a chronic acid load. Whether this also implies important metabolic complications for the patient depends on the specific patient (comorbidities) and bowel segment used [13]. Reduced kidney function increases the risk for metabolic acidosis. Colonic segments seem to be more prone to these metabolic changes as compared to ileal reservoirs. Some authors advocate the use of ileal segments in patients with impaired renal function [3]. The metabolic challenge of an ileal conduit to the patient is off course smaller than that of a reservoir because of shorter bowel segments used and because the conduit does not function as a reservoir. This hyperchloremic metabolic acidosis is subclinical in almost all cases. After a median followup of 1 year, however, 10% of patients with an ileal conduit have been reported to have a clinically important metabolic acidosis [13]. In severe cases this can result in muscle weakness and bone demineralization. In prospective series, the rate of metabolic acidosis in continent diversion and orthotopic bladder replacement varies between 26% and 45%. The definition of metabolic acidosis is not universally equal. A venous sample bicarbonate level of less than 21 mmoL/L is a frequent definition [14]. Alkalinizing therapy with oral sodium bicarbonate (1 to 2 g t.i.d.) is an effective treatment in restoring normal acid-base balance, but flatulence may decrease tolerance for this therapy. Sodium citrate (1 to 3 g q.i.d.) is a valuable alternative, but has got a bad taste. When sodium loads are to be avoided (fluid retention/pulmonary edema, hypertension), nicotinic acid (500 Mg q.d. to 2 g q.d. extended release tablets) or chlorpromazine (25 to 50 Mg q.i.d.) can decrease the need for alkalinizing agents, through inhibition of cyclic AMP-mediated chloride ion transport [15, 16].

## 5. Electrolyte Abnormalities

Associated electrolyte abnormalities may include hypokalemia, hypocalcaemia, and hypomagnesaemia. Hypokalemia is caused by both intestinal loss (secretion) and by renal wasting. It is important to realize the presence of depleted body potassium in patients with urinary diversion, since correction of acidosis can result in further potassium depletion. Probably potassium depletion is more important in patients with colonic segment diversions [17]. Clinically, this can become apparent by muscle weakness. Several case reports of patients presenting with general muscle weakness, mistaken for Guillain-Barré syndrome, after ureterosigmoïdostomy are reported in the literature [18–20]. One should therefore not forget to supplement potassium (potassium citrate 15 meq (approximately 1.6 g) b.i.d. to q.i.d.) when correcting acidosis in urinary diversion [21]. 

Hypocalcaemia in urinary diversion is caused by renal wasting and by depletion of body calcium stores. The chronic metabolic acidosis is continuously buffered by bone carbonate. Mobilization of carbonate results in calcium release from the bone. The excess of calcium is cleared by the kidneys, where the presence of acidosis and of sulfate further inhibits calcium reabsorption. Calcium supplements (500 Mg to 1 g q.d.) are the treatment of choice.

Hypomagnesaemia is a rare condition. Renal wasting may occur but nutritional depletion often plays a role as well.

## 6. Calculus Formation

The incidence of renal stone formation increases in patients with intestinal urinary diversion. After 20 years of followup, up to 20% of patients with ileal conduits will have renal calculi [22]. At the metabolic level, the presence of hyperchloremic metabolic acidosis results in calcium phosphate and/or calcium oxalate stone formation. The alkalic urine with increased levels of urinary phosphate, sulfate, and magnesium and decreased levels of urinary citrate is susceptible for stone formation. The presence of foreign materials (such as sutures and staples) will additionally act as a nidus. Intestinal mucus can also function as a nidus for calcifications as well as a harbor for chronic infection. Pouch calculi are reported in about 10% of patients with continent diversion. Initial reports mentioned up to 25% of calculi in certain pouches. Probably exposed staples in these techniques were responsible for these very high rates [23–25]. The number of calculi in orthotopic neobladders is generally lower. Chronic colonization/infection of the diversion, especially with urease producing bacteria, will result in struvite and/or carbonate apatite stones.

## 7. Vitamin B12

Vitamin B12 absorption occurs in the terminal ileum. The use of this segment in urinary diversion can result in vitamin B12 deficiency. Since body stores of vitamin B12 are sufficient for 3 to 5 years, deficiency will only become apparent after several years. Therefore, one should obtain vitamin B12 levels within this time frame. Clinically, deficiency can result in insidious but irreversible neurological deficits and megaloblastic macrocytic anemia. Radiotherapy may predispose patients to this kind of malabsorption [26]. When vitamin B12 deficiency is suspected, supplementation should be started. Normal serum vitamin B12 levels do not always exclude deficiency. Oral supplementation with high doses (1 to 2 g q.d.) might be as effective as parenteral (intramuscular or subcutaneous) administration (1 g monthly) [27].

## 8. Bone Metabolism

The major effect of urinary diversion on bone metabolism is demineralization. The process of demineralization can be explained by several pathways. The chronic metabolic hyperchloremic acidosis is buffered by bone minerals. Mobilization of calcium, carbonate, and sodium results in demineralization. Secondly, acidosis impairs renal activation of vitamin D. Activated vitamin D is necessary for normal bone mineralization. Acidosis also activates osteoclasts, resulting in bone resorption. Due to the use of bowel segments in urinary diversion, intestinal absorption of calcium and vitamin D can also be impaired. Parathormone does not seem to play a role in demineralization after urinary diversion. Again, patients with decreased renal function are particularly at risk for this sequence. Severe bone demineralization leading to osteomalacia in adults or rickets in children is rare. In these bone diseases, bone minerals are replaced by osteoid. Clinically, it results in pain in the weight bearing bones. More subtle changes in bone mineralization are probably present in the majority of patients with urinary diversion for extended periods. It is not clear to which extent patients should be screened (repeated bone densitometry) or to which extent prevention should be undertaken in risk groups (such as perimenopausal women, children, and patients with impaired kidney function). Treatment of metabolic acidosis by oral sodium bicarbonate and administration of vitamin C supplements are able to avoid bone demineralization [28, 29]. In severe cases, supplemental vitamin D and calcium could be necessary to improve bone remineralization.

## 9. Hepatic Metabolism

In normal circumstances, hepatic metabolism is not significantly altered by urinary diversion. Due to increased ammonia reabsorption from urine in the bowel segments, the liver receives an increased ammonia load. The liver uses ammonia in the ornithine cycle to create urea, which is in turn secreted trough the kidneys. The normal liver can adapt easily to this increased load. Infection with urea splitting bacteria (Proteus mirabilis, Klebsiella oxytoca, etc.) further increases the ammonia load in an acute way. In addition, endotoxins significantly affect hepatic transport and metabolism [30]. Certainly in the case of urinary obstruction, such an infection can lead to hyperammonemic encephalopathy and even to hepatic coma [31]. Treatment consists of drainage of the diversion and antibiotics. Limited protein intake, the use of nonabsorbable disaccharides (e.g., lactulose enemata), and oral neomycin can diminish the nitrogen load for the patient. Preexisting hepatic disease obviously will put the patient at risk for this kind of complication. It is the most frequent cause of altered sensorium in patients with urinary diversion. In patients with noncontinent diversion such as an ileal conduit, the occurrence of these complications is extremely rare.

## 10. Renal Function

The normal glomerular filtration rate (GFR) for adults is approximately from 100 to 130 ml/min/1,73 m². After the age of 40, it decreases progressively with approximately 1 ml/min/1,73 m² per year. Factors that impair renal function after urinary diversion are obstruction of the ureters (stenosis of uretero-intestinal anastomosis), recurrent infection, and urinary lithiasis. The exact impact of urinary diversion on renal function is not known [14]. It has been shown that GFR decreases 15–25% after urinary diversion with a followup of 11 years. It is important to diagnose reversible causes of upper urinary tract deterioration and to treat these causes accordingly. It is equally important to know the level of renal function prior to urinary diversion, as this may have an impact on future decisions. Long-term monitoring of renal function, at least annually, is advisable after any form of urinary diversion. Serum creatinine is not a sensitive parameter to follow the renal function. The use of renal ultrasound together with serum creatinine is to be considered a screening method of the upper urinary tract. When in doubt, nuclear scans to determine the GFR or diuretic renograms should be performed.

## 11. Abnormal Drug Kinetics

Many substances are secreted in urine. In case of urinary diversion, they can be reabsorbed by the intestinal segments that are incorporated in the urinary tract. This might have consequences for diagnostic purposes. Urine glucose screening in diabetes patients might be inaccurate due to glucose absorption. Therefore, blood tests should be performed for surveillance [32]. On the therapeutic level, mainly drugs that are secreted unchanged in urine and absorbed by the intestine can cause problems. Methotrexate causes toxicity in patients with ileal conduits [33, 34]. When chemotherapy is given to patients with continent diversion, an indwelling catheter should be placed during treatment. Also other drugs such as phenytoin, theofyllin, lithium, and several antibiotics are known to be reabsorbed in the intestinal segments [35–37]. The clinical significance of this process is difficult to summarize in general terms, since an important interindividual variability in ileal absorption is present [38]. A clinician should be aware of the need for dose adaptations in certain clinical settings.

## 12. Conclusion

Urinary diversion is performed frequently in current urological practice. When a diversion is carried out, the patient will undergo metabolic changes. Depending on the bowel segment used, the length of the bowel segment in the type of diversion, these metabolic consequences will be more or less pronounced. An ileal conduit is the diversion of choice when the metabolic changes want to be kept to a minimum. Even this group of patients will have lower bicarbonate levels and will have episodes of severe acidosis. Continent urinary diversion (cutaneous or neobladders) will result in longer contact between urine and intestinal segments. These patients will require sodium bicarbonate substitution. Life-long followup of patients with urinary diversion is mandatory, not only from oncological but also from metabolic perspective. It is unclear whether patients should be screened for bone health but one should be aware of increased risk in certain patient groups.

## References

[1] Bricker EM (1950). Bladder substitution after pelvic evisceration. *The Surgical Clinics of North America*.

[2] Schmidt JD, Hawtrey CE, Flocks RH, Culp DA (1973). Complications, results and problems of ileal conduit diversions. *Journal of Urology*.

[3] Hautmann RE, Miller K, Steiner U, Wenderoth U (1993). The ileal neobladder: 6 Years of experience with more than 200 patients. *Journal of Urology*.

[4] Hautmann RE (2010). Surgery illustrated—surgical atlas ileal neobladder. *British Journal of Urology International*.

[5] Studer UE, Varol C, Danuser H (2004). Surgical Atlas Orthotopic ileal neobladder. *British Journal of Urology International*.

[6] Joniau S, Benijts J, Van Kampen M (2005). Clinical experience with the N-shaped ileal neobladder: assessment of complications, voiding patterns, and quality of life in our series of 58 patients. *European Urology*.

[7] Ghoneim MA, Shaaban AA, Mahran MR, Kock NG (1992). Further experience with the urethral Kock pouch. *Journal of Urology*.

[8] Thüroff JW, Riedmiller H, Fisch M, Stein R, Hampel C, Hohenfellner R (2010). Mainz pouch continent cutaneous diversion. *British Journal of Urology International*.

[9] Thuroff JW, Alken P, Engelmann U (1985). The Mainz pouch (mixed augmentation ileum ’n Zecum) for bladder augmentation and continent urinary diversion. *European Urology*.

[10] Rowland RG, Mitchell ME, Bihrle R (1987). Indiana continent urinary reservoir. *Journal of Urology*.

[11] Rowland RG (1996). Present experience with the Indiana pouch. *World Journal of Urology*.

[12] McDougal WS (1986). Bladder reconstruction following cystectomy by uretero-ileo-colourethrostomy. *Journal of Urology*.

[13] Kamidono S, Oda Y, Ogawa T (1985). Clinical study of urinary diversion. II: review of 41 ileocolic conduit cases, their complications and long term (6~9 years) follow-up. *Nishinihon Journal of Urology*.

[14] Bakke A, Jensen KM, Jonsson O (2007). The rationale behind recommendations for follow-up after urinary diversion: an evidence-based approach. *Scandinavian Journal of Urology and Nephrology*.

[15] Koch MO, McDougal WS (1985). Chlorpromazine: adjuvant therapy for the metabolic derangements created by urinary diversion through intestinal segments. *Journal of Urology*.

[16] Koch MO, McDougal WS (1985). Nicotinic acid: treatment for the hyperchloremic acidosis following urinary diversion through intestinal segments. *Journal of Urology*.

[17] Williams RE, Davenport TJ, Burkinshaw L, Hughes D (1967). Changes in whole body potassium associated with uretero-intestinal anastomoses. *British Journal of Urology*.

[18] Valtier B, Mion G, Pham LH, Brochard L (1989). Severe hypokalaemic paralysis from an unusual cause mimicking the Guillain-Barre sydrome. *Intensive Care Medicine*.

[19] Van Bekkum JW, Bac DJ, Nienhuis IE, De Leeuw PW, Dees A (2002). Life-threatening hypokalaemia and quadriparesis in a patient with ureterosigmoidostomy. *Netherlands Journal of Medicine*.

[20] Rafique M (2006). Life threatening hypokalemia and quadriparesis in a patient with ureterosigmoidostomy. *International Urology and Nephrology*.

[21] Koff SA (1975). Mechanism of electrolyte imbalance following urointestinal anastomosis. *Urology*.

[22] McDougal WS, Koch MO (1991). Impaired growth and development and urinary intestinal interposition. *The American Association of Genitourinary Surgeons*.

[23] Holmes DG, Thrasher JB, Park GY, Kueker DC, Weigel JW (2002). Long-term complications related to the modified Indiana pouch. *Urology*.

[24] Skinner DG, Lieskovsky G, Boyd SD (1987). Continuing experience with the continent ileal reservoir (Kock pouch) as an alternative to cutaneous urinary division: an update after 250 cases. *Journal of Urology*.

[25] Terai A, Ueda T, Kakehi Y (1996). Urinary calculi as a late complication of the Indiana continent urinary diversion: comparison with the kock pouch procedure. *Journal of Urology*.

[26] Kinn AC, Lantz B (1984). Vitamin B12 deficiency after irradiation for bladder carcinoma. *Journal of Urology*.

[27] Vidal-Alaball J, Butler CC, Cannings-John R (2005). Oral vitamin B12 versus intramuscular vitamin B12 for vitamin B12 deficiency. *Cochrane Database of Systematic Reviews*.

[28] Stein R, Fisch M, Andreas J, Bockisch A, Hohenfellner R, Thüroff JW (1998). Whole-body potassium and bone mineral density up to 30 years after urinary diversion. *British Journal of Urology*.

[29] McDougal WS, Koch MO, Shands C, Price RR (1988). Bony demineralization following urinary intestinal diversion. *Journal of Urology*.

[30] McDougal WS, Heimburger S, Wilmore DW, Pruitt BA (1978). The effect of exogeneous substrate on hepatic metabolism and membrane transport during endotoxemia. *Surgery*.

[31] Albersen M, Joniau S, Van Poppel H, Cuyle PJ, Knockaert DC, Meersseman W (2007). Urea-splitting urinary tract infection contributing to hyperammonemic encephalopathy. *Nature Clinical Practice Urology*.

[32] Sridhar KN, Samuell CT, Woodhouse CRJ (1983). Absorption of glucose from urinary conduits in diabetics and non-diabetics. *British Medical Journal*.

[33] Fossa SD, Heilo A, Bormer O (1990). Unexpectedly high serum methotrexate levels in cystectomized bladder cancer patients with an ileal conduit treated with intermediate doses of the drug. *Journal of Urology*.

[34] Bowyer GW, Davies TW (1987). Methotrexate toxicity associated with an ileal conduit. *British Journal of Urology*.

[35] Ekman I, Mansson W, Nyberg L (1989). Absorption of drugs from continent caecal reservoir for urine. *British Journal of Urology*.

[36] Davidsson T, Åkerlund S, Whitef T, Olaisson G, Månsson W (1996). Mucosal permeability of ileal and colonie reservoirs for urine. *British Journal of Urology*.

[37] Alhasso A, Bryden AA, Neilson D (2000). Lithium toxicity after urinary diversion with ileal conduit. *British Medical Journal*.

[38] Mattioli F, Tognoni P, Manfredi V (2006). Interindividual variability in the absorption of ciprofloxacin and hydrocortisone from continent ileal reservoir for urine. *European Journal of Clinical Pharmacology*.

